# Patient-Reported Outcome (PRO) as an Addition to Long-Term Results after High-Precision Stereotactic Radiotherapy in Patients with Secreting and Non-Secreting Pituitary Adenomas: A Retrospective Cohort Study up to 17-Years Follow-Up

**DOI:** 10.3390/cancers11121884

**Published:** 2019-11-27

**Authors:** Kerstin A. Kessel, Christian D. Diehl, Markus Oechsner, Bernhard Meyer, Jens Gempt, Claus Zimmer, Friederike Schmidt-Graf, Stephanie E. Combs

**Affiliations:** 1 Department of Radiation Oncology, Technical University of Munich (TUM), 81675 Munich, Germany; christian.diehl@tum.de (C.D.D.); markus.oechsner@mri.tum.de (M.O.); Stephanie.Combs@tum.de (S.E.C.); 2 Institute of Radiation Medicine (IRM), Helmholtz Zentrum München, 85764 Neuherberg, Germany; 3 Deutsches Konsortium für Translationale Krebsforschung (DKTK), DKTK Partner Site, 81675 Munich, Germany; Bernhard.Meyer@tum.de (B.M.); Jens.Gempt@tum.de (J.G.); Claus.Zimmer@tum.de (C.Z.); 4 Department of Neurosurgery, Technical University of Munich (TUM), 81675 Munich, Germany; 5 Department of Neuroradiology, Technical University of Munich (TUM), 81675 Munich, Germany; 6 Department of Neurology, Technical University of Munich (TUM), 81675 Munich, Germany; f.schmidt-graf@tum.de

**Keywords:** pituitary adenoma, stereotactic fractionated radiotherapy, patient-reported outcome, long-term survival, toxicity, secreting

## Abstract

High-precision radiotherapy has been established as a valid and effective treatment option in patients with pituitary adenomas. We report on outcome after fractionated stereotactic radiotherapy (FSRT) in correlation with patient-reported outcomes (PROs). We analyzed 69 patients treated between 2000 and 2019. FSRT was delivered with a median total dose of 54 Gy (single fraction: 1.8 Gy). PRO questionnaires were sent to 28 patients. Median overall survival was 17.2 years; mean local control was 15.6 years (median not reached). Median follow-up was 5.8 years. Twenty (71%) patients participated in the PRO assessment. Physicians reported symptoms grade ≥3 in 6 cases (9%). Of all, 35 (51%) patients suffered from hypopituitarism at baseline, and during follow-up, new or progressive hypopituitarism was observed in 11 cases (16%). Patients reported 10 cases of severe side effects. Most of these symptoms were already graded as CTCAE (Common Terminology Criteria for Adverse Events) grade 2 by a physician in a previous follow-up exam. PROs are an essential measure and only correlate to a certain extent with the physician-reported outcomes. For high-precision radiotherapy of pituitary adenomas, they confirm excellent overall outcomes and low toxicity. In the future, the integration of PROs paired with high-end treatment will further improve outcomes.

## 1. Introduction

Pituitary adenomas are benign neoplasms of the anterior pituitary gland. These lesions account for approximately 10–15% of all intracranial neoplasms [1,2] and are classified by size, secretory properties, and cells of origin. Lesions smaller than 1 cm are described as microadenomas, and adenomas extending 1 cm or 4 cm as macroadenomas or giant adenomas, respectively [3]. Adenomas are frequently diagnosed in asymptomatic patients who have undergone magnetic resonance imaging (MRI) for unrelated reasons; hence, these lesions are named incidentalomas [4]. Approximately one-third of pituitary adenomas are non-functioning, meaning without secretion of hormones. Functioning adenomas produce hormones depending on the cells of origin. Prolactinomas are the most frequent types, followed by somatotroph, adrenocorticotroph, and thyreothroph adenomas. Only a small minority of gonadotroph adenomas are functioning [5]. Non-functioning adenomas are mostly asymptomatic until significant enlargement, and extrasellar growth causes mass-effect with visual field defects like bi-temporal hemianopsia secondary to compression of the optic chiasm, headaches, and hypopituitarism [6,7]. Secretory adenomas can cause distinct syndromes due to hypersecretion of the corresponding hormone, such as secondary amenorrhea, infertility, and gynecomastia in prolactinomas, acromegaly with somatotroph adenomas or Cushing’s disease in corticotroph adenomas [8].

Generally, treatment of pituitary adenomas is intricate and depends on the size, anatomical extension, secreting or non-secreting nature, as well as patient performance status and patient’s preference. Since the incidence of pituitary adenomas is comparably low, patients should be assigned to specialized centers offering advanced neurosurgery, experienced endocrinology, as well as advanced radiotherapy (RT) expertise [4,9,10,11]. Treatment strategies compromise active surveillance, medical treatment, radiotherapy (RT), and surgery, the latter as the first choice for large lesions causing visual field defects [12,13]. The trans-phenoidal approach is standard of care, large or giant adenomas may need an additional trans-cranial pterional access for complete removal [14]. Cranio cerebral fluid leakage and post-interventional hypopituitarism are the most common complications [15]. In some cases, depending on anatomical location, such as infiltration of the cavernous sinus, complete surgical resection remains challenging, and planned partial resection with additional RT is a reasonable approach. Post-surgically, patients with secreting pituitary adenomas often require medical treatment to normalize hormonal values [3].

The pharmacological approach is another cornerstone in the management of functioning adenomas: Dopamine antagonists such as cabergoline or bromocriptine have anti-tumor effects and are first-line treatments for prolactinomas, and somatostatin analogons like octreotide are applied for somatotroph adenomas secondary to surgery or RT with persistently elevated hormone levels [16]. Other drugs, like ketoconazole, to inhibit adrenal steroidogenesis in Cushing’s disease act asymptomatic without direct effect to the adenoma.

Stereotactic radiosurgery (SRS) or fractionated stereotactic radiotherapy (FSRT) have been established as a valid and highly effective treatment option in patients with pituitary adenomas. The concern of long-term side effects has been the main argument against early RT; however, since SRS and FSRT are available in increasing specialized centers, long-term data are becoming available, demonstrating excellent outcomes [10,17,18,19].

Indication for RT is generally seen for tumor progression if surgical resection is not feasible or has been performed several times, followed by tumor recurrence. Additionally, partial resection can be an indication for RT, especially if the tumor remnant is progressive. In hormone-secreting adenomas, RT is indicated for persistent hormonal secretion even after macroscopic total resection. High-precision RT offers the benefit of high local dose deposition and sparing of healthy tissue. Excellent results have been reported with SRS for smaller lesions, and with FSRT for larger and complex lesions [9,20,21]. Vast experienced with gamma knife radiosurgery (GKS) is available for tumors of all sizes and complement SRS and FSRT techniques with photons well [19,22,23,24]. Early data for proton therapy, which is characterized by an inverted dose profile and thus more pronounced sparing of healthy tissue, are available and very much comparable to advanced photon or GKS data [25,26].

To date, all clinical results reported include toxicity but not patient-reported outcomes (PRO), which are becoming more and more relevant since modern documentation methods and advanced communication with patients via online tools such as app-based and web-based become available [27,28,29,30,31].

The present study aims to analyze overall survival (OS) and local control (LC) after high precision photon RT, as well as long-term toxicity rates after treatment. It is one of the first studies reporting outcomes in correlation with PRO up to 17 years after treatment, and therefore complements the existing literature with novel data.

## 2. Patients and Methods

### 2.1. Patients

We analyzed 69 patients with pituitary adenoma consecutively treated between 2000 and 2019 at the Department of Radiation Oncology at the Klinikum rechts der Isar, Munich. Exclusion criteria were early termination of treatment or re-irradiation due to local recurrence. Patient data were collected prospectively and documented in the institutional database. For patient characteristics, see Table 1.

The local ethics committee of the Medical Faculty of the Technical University Munich (TUM) approved the study, vote number 407/19 S (NOSY Study).

### 2.2. Treatment

All patients were resected at least once during their treatment period. Primary radiotherapy was delivered postoperatively in an additive setting in 12 (17%) cases, and the other 57 (83%) were treated due to recurrent tumor growth. Treatment planning was performed using a stereotactic treatment setup with a thermoplastic mask system (Brainlab, Munich, Germany) by robotic ExacTrac positioning (Brainlab, Munich, Germany). An advanced radiation oncologist delineated gross tumor volume (GTV) on contrast-enhanced computed tomography (CT) and fused T1-weighted magnetic resonance imaging MRI. The clinical target volume (CTV) resulted from the GTV with an additional margin of 1–2 mm. Another 1–2 mm were added for the planning target volume (PTV). 

We performed a linear accelerator-based high-precision fractionated stereotactic radiotherapy (FSRT) with a median total dose of 54 Gy (range: 45–54 Gy) to the 95%-isodose level in single fractions of 1.8 Gy. Patients were treated five times a week, with daily image-guidance (IGRT).

### 2.3. Follow-Up and Patient-Reported Outcome (PRO)

Clinical neurological assessment, including contrast-enhanced MRI, was scheduled initially 4–6 weeks after treatment. Patients were monitored every 3–6 months in the first year and then annually in the years thereafter, or earlier in case of new symptoms or recurrent tumor on follow-up imaging. Toxicity before and after RT was rated according to the Common Terminology Criteria for Adverse Events (CTCAE, v. 5.0).

As pituitary adenomas are benign, patients tend to get lost to follow-up as they feel healthy and have no more symptoms. We established a PRO assessment via mail to gain long-term information [27,28]. At the time of analysis, 16 (23%) patients were diseased, 18 (26%) were at the clinic for a regular follow-up visit within the last year, and 7 (10%) were living abroad and counted as lost to follow-up. For the present analysis, questionnaires were sent to the remaining 28 (41%) patients. It contained questions about current symptoms, last follow-up imaging, and its findings, as well as follow-up treatments. Symptoms could be reported in four stages: None, mild, moderate, severe (see Supplementary Material S1).

Local failure was defined as re-growth of the original tumor confirmed on MRI by a senior neuroradiologist. Salvage treatment was re-resection or re-irradiation based on interdisciplinary consent in the neurooncology board. 

### 2.4. Statistics

Statistical calculations were performed using SPSS Statistics v25 (IBM, Armonk, NY, USA). We calculated OS from the end of treatment until death or last follow-up, and LC from the end of treatment until the date of local failure or until death or last follow-up. Outcome analyses were based on the Kaplan–Meier and Cox regression methods.

## 3. Results

### 3.1. Outcome

At the time of analysis, 16 (23%) patients were deceased. Reasons for death were not evaluated. Median OSwas 17.2 years (95% confidence interval (CI): 9.0–25.4). Local failure was reported in 6 (9%) cases and resulted in a mean LC of 15.6 years (95% CI: 14.2–17.0, the median was not reached), see Table 2. OS and LC were not influenced by hormone functioning (OS: *p* = 0.962, LC: *p* = 0.576) or RT type (OS: *p* = 0.719, LC: *p* = 0.896). None of the patients developed a histopathological change to malignant pituitary disease after irradiation.

### 3.2. Toxicity

Median follow-up was 5.8 years (range: 0.1–17.4 years). We contacted 28 patients for PRO assessment, of which 20 (71%) participated via mail. The median time between treatment and PRO was 13.3 years (range: 5.3–17.4 years).

Table 3 reports on all new and worsened toxicity before RT and during follow-up according to the CTCAE classification. Before RT, 5 (7%) patients were already blind, and 3 (4%) presented with severe visual deficits (e.g., diplopia, hemianopia, visual field defects). Overall toxicity rates after RT were very low. No radiation-induced brain necrosis was reported. Symptoms ≥ grade 3 were reported in 6 cases (9%): Five patients developed vision loss or visual deficits, and one patient severe sensory disorders due to a secondary disease of multiple sclerosis. Of all, 35 (51%) patients suffered from hypopituitarism at baseline, and during follow-up, new or progressive hypopituitarism was observed in 11 cases (16%). Elevated hormonal levels resulting from uncontrolled pituitary gland function were seen in 5 (7%) patients as acromegaly or Cushing’s syndrome. Common acute side effects after RT were fatigue (11, 19%), hair loss (14, 24%), headache (9, 16%), and dizziness (8, 14%); however, all were mild (grade 1 or 2) and improved in most cases during follow-up.

**Figure 1:**
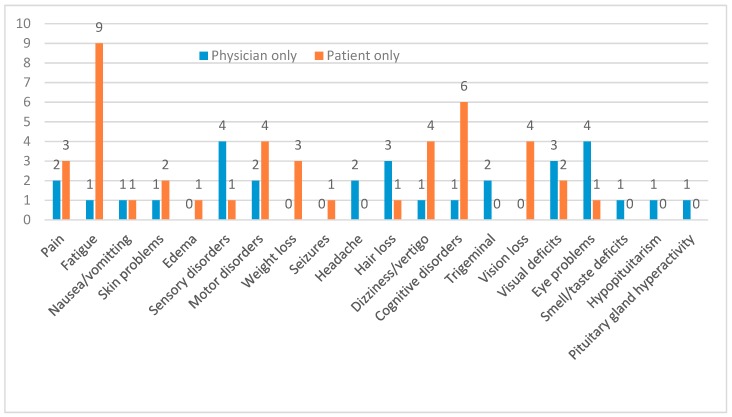
Differences in physician-reported outcome and patient-reported outcome (PRO). Symptoms are listed that were reported by either physicians only during follow-up, or patients only during PRO assessment.

PRO data are listed separately and not included in the physician-reported toxicity. Table 4 and Figure 1 show only the results of the PRO. Patients reported 10 cases of severe side effects. One patient developed new severe skin erythema, and two patients reported new severe motor deficits. In all other cases, the symptom was already graded as CTCAE grade 2 by a physician in a previous follow-up exam.

## 4. Discussion

Long-term evaluation of high-precision RT as FSRT demonstrates excellent local control; treatment outcome is well tolerated by all patients, which is in part due to very low side effects. PROs confirm convincing long-term outcomes and low side effects. For the first time, this analysis reports PROs for high-precision RT of patients with pituitary lesions.

Generally, interdisciplinary discussion of patients with pituitary tumors is necessary. While neurosurgical resection remains to be the mainstay of treatment, outcome data from stereotactic radiotherapy delivered as SRS or FSRT convincingly and reproducibly show excellent outcomes in terms of local control and toxicity.

In the present study, we found a 5-year and 10-year LC of 92% and 87%, respectively, which is in accordance with previously published data. While the 5-year local control after FSRT is between 90% and 95%, few groups have reported on long-term outcome. The study reported by Weber and colleagues published 27 patients treated with either adjuvant or radical, with a median follow-up of six years; local control rates were >95% at five years [20]. Sun et al. reported 33 patients with pituitary adenomas with local control of 94% at 36 months [9]. A long-term follow-up of patients treated in Heidelberg revealed local control of 90.4% at 10 years and thus offering long-term follow-up; there was no difference between patient sex or the secreting or non-secreting nature of the disease [10]. When closely looking at the local control curves, one notices a decline in local control at 20 years after RT. Therefore, while being a benign tumor, long-term follow-up, including endocrinological assessment as well as imaging, is mandatory. 

In our analyses, we did not find a correlation with hormone functioning and LC or OS. However, Langsenlehner et al. showed inferior local control of non-secreting adenomas [18]. The reason for this is mostly unclear, some arguing that secreting adenomas are often treated with higher total doses, or it may depend on hormone-subtype secreted: Patients with pituitary adenomas secreting somatotropin are generally associated with a better prognosis, whereas prolactinomas are associated with worse outcome [32,33]. This may be due to different dose regimes with higher doses delivered to functioning adenomas. 

In a group of 92 patients, improved quality of life (QOL) was reported by 24%, and stabilization of QOL was noted by 68% of all patients [10]. The same report showed a negative impact on QOL in only 5%, while 95% of all patients assessed RT to have preserved QOL or even improved QOL. Van Beek et al. also reported low rates of side effects, and additionally, they found no significant difference in QOL or cognition in patients undergoing postoperative irradiation vs. those who underwent surgery alone [34].

While FSRT offers the radiobiological benefits of fractionation, several experienced centers argue for radiosurgery in pituitary adenomas. Stereotactic Radiosurgery (SRS) can be delivered either with specialized machines such as the gamma knife or cyberknife system, or with dedicated linear accelerators. Overall, the GKS technology offers the most precise dose deposition with the best sparing of healthy tissue. In a retrospective analysis from three large Korean centers, long-term data of GKS on local control and acromegaly were reported by Kong et al. [19]. The study included 138 patients; after a mean follow-up of 85.2 months, time to the endocrine remission and control under long-acting somatostatin analogues was 138 months and 96 months, respectively. Favorable factors for outcome were female sex, normal age-adjusted insulin growth factor-1/2, and GKS as an adjuvant treatment were significantly favorable factors for remission. Very few patients developed radiosurgery-related hypopituitarism. Hong and colleagues reported on 289 patients with prolactinomas treated with GKS at ten international centers [35]; the endocrine remission rates were 28%, 41%, and 54% at 3, 5, and 8 years, respectively, after SRS. In that study, however, 25% of all patients reported new treatment-related hormonal deficiencies and 3% of all patients developed visual complications related to SRS.

In the present analyses of long-term toxicity, vision loss and visual deficits were the most reported symptoms by both physicians and patients. Physicians reported 27 cases of grade 1/2 symptoms and 5 cases of grade 3. Accordingly, patients reported optic symptoms in 14 cases graded from mild to severe. Considering the close anatomical relation of pituitary tumors to the visual apparatus, including the optic nerves and chiasm, special attention must be given to dose to those organs at risk (OARs). Dose-response modeling has shown that the Dmax should be limited to 12 Gy in 1 fraction based on QUANTEC data [21], while older considerations have argued for smaller single doses [36]. Most radiosurgery centers were conservative in doses to the optic structures, and their data confirmed that radiation-induced optic neuritis could be kept at 2% or lower with patients receiving more than 8 Gy to a short segment of the optic apparatus [37]. However, it must be kept in mind that optic nerve motion during treatment can be substantial, and that most calculated dose distributions based on static CT imaging may underestimate dose to the optic OAR [38]. 

Documentation of long-term side effects remains a challenge, and objective assessment may not correlate with patient-centered assessment. Therefore, PROs are becoming continuously more important in follow-up care due to increasing evidence that tight connection with patients, integration of PRO into care plans, as well as the use of electronic devices such as apps improves outcomes. Recently, Denis et al. [39] showed in an analysis of lung cancer patients that regularly reported symptoms lead to a 6-month longer OS. Basch et al. [40] performed a randomized study with 766 patients. The intervention group regularly reported PRO data, which resulted in an improved OS as well. 

As with all retrospective analyses, the main problem is heterogeneous and incomplete data. PRO data are also a relevant supplement that leads to greater completeness of data and should not be seen as an alternative. PRO is especially useful in cancer entities were long-term results are of interest and patients are lost to follow-up. In our analysis, the median time between treatment and PRO was 13.3 years. Hence, PRO is an easily applicable tool for long-term follow-up, generating valuable data on QOL and late effects years after RT for pituitary adenomas. In this study, 20 of 28 patients (71%) participated in PRO, implying a fairly high response rate partially many years after treatment. This reflects the favorable course of the disease and long-term preservation of good health of patients with pituitary adenomas treated with RT.

The interpretation of PRO data is still difficult. In our study, we saw several symptoms graded as severe by the patients. However, this is not comparable to a CTCAE severe symptom grade 3. The translation of patient-reported and physician-reported symptoms is an existing limitation also in this study [41]. As shown by other groups, symptoms are usually graded higher by patients than by physicians. The most discrepancies exist for symptoms where patients feel the most personal impact, e.g., alopecia, headache [42], or for conditions that cannot be measured objectively like pain and fatigue. As shown in Figure 1, fatigue is the most reported long-term symptom by patients. It can be secondary to hormone deficits, anemia, or infections that have to be ruled out by a physician. Otherwise, it should be seen as a long-term effect after RT, hence physical activity should be recommended during follow-up visits as generally acknowledged countermeasures. 

Vision loss and visual deficits are a severe complication in the treatment of pituitary adenomas, which is luckily a very rare phenomenon in the era of high precision medicine. However, depending on the size of the lesion, pretreatment damage, and other patient-related factors, some visual decline can be possible. In the PRO assessment, we observed six patients who newly reported mild-to-moderate deficits; however, no objective data on eye-sight and visual field were included in the PRO documentation. Any patient reporting on sight deficits should be taken seriously and must be seen by an ophthalmologist, and/or cranial MRI must be performed to rule out a recurrent tumor or other medical conditions. Therefore, the identification of such symptoms early by PRO will help reverse or stop the deterioration of tumor or treatment complications and help identify tumor recurrences very early when symptoms begin to develop. Further long-term symptoms only reported by PRO were cognitive disorders and dizziness. This might be due to the increasing age of patients and not caused by RT treatment. However, prospective studies must be implemented to provide evidence. 

There is an increasing effort to establish a PRO-CTCAE classification by the National Cancer Institute (NCI) to improve symptom comparison [43,44]. The routine implementation into the clinical workflow must still be performed. The interpretation also becomes more difficult if elderly patients are reporting with increasing comorbidities [27]. However, the need for permanent close PRO monitoring is obvious, and in the future, the assessment will be done electronically. In our previous works, we could show that both patients and physicians are willing to participate via app-/web-based tools [29,30,31]. 

The integration of digital information available from computed tomography (CT) and/or MRI may also enable prediction of treatment outcome, identification of early responders, or predictors of treatment failure. Fan et al. [45] demonstrated that incorporating an MRI-based radiomics signature and clinical features into a radiomics model improved the accuracy of RT response prediction in patients with acromegaly. The authors could show that the radiomics model had a higher rate of response prediction, which could potentially improve treatment strategies for patients with acromegaly. 

## 5. Conclusions

PROs are an essential measure and only correlate to a certain extent with the physician-reported outcomes. For high-precision RT of pituitary lesions, they confirm excellent overall survival, low recurrence rates, and toxicity. PRO will help to foster patient–physician connection and will lead to early detection of side effects or tumor recurrences when symptoms and/or changes in QOL, patient lifestyle, resilience, and stamina are observed more promptly. In the future, standardized integration of PRO paired with high-end treatment will further improve outcomes and enhance patient care in modern radiation oncology.

## Figures and Tables

**Table 1:** Patient characteristics.

Characteristic	Specification	*N* (%)	Median (Range)
Gender	Female	30 (44%)	
	Male	39 (56%)	
Age at RT [years]			61 (28–87)
Size	Macroadenoma	66 (96%)	
	Microadenoma	3 (4%)	
Immunohistochemical classification	PRL	7 (10%)	
	GH	5 (7%)	
	ACTH	5 (7%)	
	PRL & GH	2 (3%)	
	FSH	2 (3%)	
	TSH	1 (1%)	
	Non-functioning	47 (68%)	
RT type	Primary treatment	12 (17%)	
	Salvage treatment	57 (83%)	
PTV [mL]			21.0 (1.9–124.5)
Total dose [Gy]			50.4 (45–54)

RT: Radiotherapy; PRL: Prolactin; GH: Growth hormone; ACTH: Adrenocorticotropic hormone; FSH: Follicle-stimulating hormone; TSH: Thyroid-stimulating hormone; PTV: Planning target volume.

**Table 2:** OS and LC life table for pituitary adenomas.

Outcome	3-year	5-year	8-year	10-year	15-year
OS	95%	90%	86%	79%	65%
LC	97%	92%	87%	87%	87%

OS: Overall survival; LC: Local control.

**Table 3:** New and worsened toxicity during follow-up (graded according to the CTCAE classification, v. 5.0). Symptoms before radiotherapy (RT) were considered as baseline. The time intervals were defined into five groups: Directly before RT, 0–6 months after RT (acute toxicity), 6–24 months after RT (medium-term toxicity), >2 years after RT (long-term toxicity). Symptoms were counted based on the number of patients observed during each interval. PRO symptoms were reported in four stages: None, mild (1), moderate (2), severe (3).

CTCAE Grade	PRE RT				<6 Months				>6 Months to <24 Months				>2 Years				PRO		
	*n* = 69				*n* = 58				*n* = 47				*n* = 57				*n* = 20		
	1	2	3	4	1	2	3	4	1	2	3	4	1	2	3	4	1	2	3
Pain	-	-	-	-	2	-	-	-	2	1	-	-	2	1	-	-	4	1	-
					(3%)				(4%)	(2%)			(4%)	(2%)			(20%)	(5%)	
Fatigue	-	-	-	-	9	2	-	-	2	-	-	-	6	7	-	-	7	5	2
					(16%)	(3%)			(4%)				(11%)	(12%)			(35%)	(25%)	(10%)
Nausea/vomiting	-	-	-	-	1	-	-	-	1	-	-	-	2	-	-	-	1	-	-
					(2%)				(2%)				(4%)				(5%)		
Skin problems/erythema	-	-	-	-	3	-	-	-	-	-	-	-	-	-	-	-	-	1	1
					(5%)													(5%)	(5%)
Edema	-	-	-	-	-	1	-	-	1	-	-	-	-	-	-	-	1	-	-
						(2%)			(2%)								(5%)		
Sensory disorders	-	1	-	-	5	-	-	-	-	2	-	-	1	5	1	-	1	-	-
		(1%)			(9%)					(4%)			(2%)	(9%)	(2%)		(5%)		
Motor disorders	1	1	-	-	1	2	-	-	2	-	-	-	1	5	-	-	2	-	2
	(1%)	(1%)			(2%)	(3%)			(4%)				(2%)	(9%)			(10%)		(10%)
Weight loss	-	-	-	-	-	-	-	-	-	-	-	-	1	2	-	-	3	-	-
													(2%)	(4%)			(15%)		
Seizures	-	-	-	-	1	-	-	-	-	-	-	-	2	1	-	-	2	-	-
					(2%)								(4%)	(2%)			(10%)		
Headache	5	3	-	-	8	1	-	-	5	-	-	-	4	1	-	-	4	-	1
	(7%)	(4%)			(14%)	(2%)			(11%)				(7%)	(2%)			(20%)		(5%)
Hair loss	-	-	-	-	14	-	-	-	-	-	-	-	-	-	-	-	3	2	-
					(24%)												(15%)	(10%)	
Dizziness/vertigo	3	1	-	-	8	-	-	-	1	2	-	-	1	3	-	-	3	2	-
	(4%)	(1%)			(14%)				(2%)	(4%)			(2%)	(5%)			(15%)	(10%)	
Cognitive disorders	1	1	-	-	-	1	-	-	-	1	-	-	6	4	-	-	5	1	
	(1%)	(1%)				(2%)				(2%)			(11%)	(7%)			(25%)	(5%)	
Trigeminal neuropathy	-	-	-	-	1	-	-	-	-	-	-	-	1	-	-	-	-	-	-
					(2%)								(2%)						
Facial neuropathy	1	-	-	-	-	-	-	-	-	-	-	-	1	1	-	-	-	-	-
	(1%)												(2%)	(2%)					
Vision loss	3	2	-	5	3	1	-	-	-	2	1	--	2	5	2	-	5	2	3
	(4%)	(3%)		(7%)	(5%)	(2%)				(4%)	(2%)		(4%)	(9%)	(4%)		(25%)	(10%)	(15%)
Visual deficits	11	8	3	-	5	3	-	-	1	1	1	-	1	3	1	-	3	1	-
	(16%)	(12%)	(4%)		(9%)	(5%)			(2%)	(2%)	(2%)		(2%)	(5%)	(2%)		(15%)	(5%)	
Eye problems	1	4	-	-	1	-	-	-	2	1	-	-	2	6	-	-	1	-	-
	(1%)	(6%)			(2%)				(4%)	(2%)			(4%)	(11%)			(5%)		
Smell/taste deficits	-	-	-	-	1	2	-	-	1	-	-	-	2	-	-	-	-	-	-
					(2%)	(3%)			(2%)				(4%)						
Hypopituitarism	-	35	-	-	-	3	-	-	-	2	-	-	-	6	-	-	-	1	1
		(51%)				(5%)				(4%)				(11%)				(5%)	(5%)
Pituitary gland hyperactivity	2	5	-	-	1	1	-	-	-	1	-	-	-	2	-	-	-	-	-
	(3%)	(7%)			(2%)	(2%)				(2%)				(4%)					

RT: Radiotherapy; PRO: Patient-reported outcome; CTCAE: Common Terminology Criteria for Adverse Events.

**Table 4:** Comparison of physician-reported outcome after RT and PRO (*n* = 20). In both groups, only new and worsened symptoms after RT are listed.

Symptom	Physician-Reported after RT		PRO	
CTCAE Grade	**1/2**	**3**	**1/2**	**3**
Pain	6	-	5	-
	(30%)		(25%)	
Fatigue	8	-	12	2
	(40%)		(60%)	(10%)
Nausea/vomiting	1	-	1	-
	(5%)		(5%)	
Skin problems/erythema	1	-	1	1
	(5%)		(5%)	(5%)
Edema	-	-	1	-
			(5%)	
Sensory disorders	3	1	1	-
	(15%)	(5%)	(5%)	
Motor disorders	2		2	2
	(10%)		(10%)	(10%)
Weight loss	-	-	3	-
			(15%)	
Seizures	1	-	2	-
	(5%)		(10%)	
Headache	5	-	4	1
	(25%)		(20%)	(5%)
Hair loss	7	-	5	-
	(35%)		(25%)	
Dizziness/vertigo	2	-	5	-
	(10%)		(25%)	
Cognitive disorders	1	-	6	-
	(5%)		(30%)	
Trigeminal neuropathy	2	-	-	-
	(10%)			
Facial neuropathy	-	-	-	-
Vision loss	3	1	7	3
	(15%)	(5%)	(35%)	(15%)
Visual deficits	5	-	4	-
	(25%)		(20%)	
Eye problems	4	-	1	-
	(20%)		(5%)	
Smell/taste deficits	1	-	-	-
	(5%)			
Hypopituitarism	3	-	1	1
	(15%)		(5%)	(5%)
Pituitary gland hyperactivity	1	-	-	-
	(5%)			

RT: Radiotherapy; PRO: Patient-reported outcome; CTCAE: Common Terminology Criteria for Adverse Events.

## Supplementary Materials

The following are available online at https://www.mdpi.com/2072-6694/11/12/1884/s1, Supplementary Material S1: Symptoms could be reported in four stages.
