# Supplementary material for: Patient-Reported Outcome (PRO) as an Addition to Long-Term Results after High-Precision Stereotactic Radiotherapy in Patients with Secreting and Non-Secreting Pituitary Adenomas: A Retrospective Cohort Study up to 17-Years Follow-Up
Source: Cancers (Basel). 2019 Nov 27;11(12):1884. doi: 10.3390/cancers11121884 (PMC6966568; doi:10.3390/cancers11121884)
Supplement: Supplementary file 1 [file cancers-11-01884-s001.pdf]

Klinikum rechts der Isar

Technische Universität München

Patienten Betreuung - Fragebogen

Name: xx xx, *xx.xx.xxxx

Aktuelles Datum: ________________________________

Aktuelle Telefonnummer: ________________________________

Letzte Kontrolluntersuchung/MRT
(Wenn möglich, legen Sie bitte den originalen Befundbericht als Kopie bei, insbesondere bei Befundverschlechterung, oder Angabe, wo wir diesen erhalten können.)

Datum:

Befundbeurteilung:

<u>Aktuelle</u> Beschwerden nach der Strahlentherapie – Kreuzen Sie bitte Zutreffendes an. Nutzen Sie bitte die Rückseite um genauere Angaben zu machen:

	Überhaupt nicht	Wenig	Mäßig	Sehr
Allgemeine Schmerzen in der bestrahlten Region?	0	1	2	3
Müdigkeit/Abgeschlagenheit?	0	1	2	3
Übelkeit?	0	1	2	3
Hautprobleme/Ausschläge in der bestrahlten Region?	0	1	2	3
Narbenbildung/Verhärtungen in der bestrahlten Region?	0	1	2	3
Pigmentierungsveränderung in der bestrahlten Region?	0	1	2	3
Schwellungen/Ödeme? Welche/Wo?	0	1	2	3
Beschwerden mit dem Sehen?	0	1	2	3
Gesichtsfeldeinschränkungen? Doppelbilder?	0	1	2	3
Taubheitsgefühle/Missempfindungen im Gesicht?	0	1	2	3
Ausfälle der Gesichtsmuskulatur?	0	1	2	3
Beschwerden beim Gehen?	0	1	2	3
Gewichtsverlust?	0	1	2	3
Krämpfe? Epileptische Anfälle?	0	1	2	3
Haarausfall?	0	1	2	3
Kopfschmerzen?	0	1	2	3
Gleichgewichtsstörungen/Schwindel?	0	1	2	3
Gedächtnisstörungen?	0	1	2	3
Veränderung der Gemütslage?	0	1	2	3
Hypophyseninsuffizienz	0	1	2	3

Sind die Beschwerden allgemein besser geworden nach der Strahlentherapie?

☐ja ☐nein
Wenn ja, welche und wie?

Sind neue Beschwerden nach der Strahlentherapie aufgetreten?

☐ja ☐nein
Wenn ja, welche und wann?

Hatten Sie weitere Behandlungen nach der Strahlentherapie bei uns ?

Operation ☐ja ☐nein
Wenn ja, wann und welche?
(Wenn möglich, legen Sie bitte den originalen Operationsbericht als Kopie bei)

Nochmalige Strahlentherapie ☐ja ☐nein
Wenn ja, wann und welche?
(Wenn möglich, legen Sie bitte den originalen Therapiebericht als Kopie bei)
